# Frontier Warriors—Combating Cholera in Rural India

**DOI:** 10.4269/ajtmh.19-0027

**Published:** 2019-05

**Authors:** Rajlakshmi Viswanathan, Anuj Kumar

**Affiliations:** Bacteriology Group, Indian Council of Medical Research-National Institute of Virology, Pune, India

In the late afternoon sunshine, they clustered around us, smiling shyly—a group of young women and a few men. We were assembled at the “Anganwadi” or courtyard shelter—a rural childcare center in the village of Rahude, a small tribal settlement in Maharashtra, a state located in western India.

A cholera outbreak had swept through the village a few weeks back. My colleague and I were here from Pune, nearly 300 km away, where we worked at the newly set up bacteriology division at the Indian Council of Medical Research-National Institute of Virology. We had confirmed cholera as the cause of an explosive outbreak of acute watery diarrhea.

Curious to know how this ancient scourge of mankind had affected the village, we had driven down to the district headquarters at Nashik. Accompanied by two local health workers, we set off for Rahude. A brief chat on the way painted a picture of a tribal settlement populated by a small community. Rahude is a tribal village, some 10 km interior to the nearest highway. A few decades ago, the area was under forest cover, which has now been cleared, as the tribal population is adapting more and more to farming. As the car took the road connecting the highway with the village, we saw farmers working in the fields with traditional farming tools.

However, farming is not the only profession. During summers, when there is no work on the farms, village males move to cities and work as contract laborers. By the time the rains start, they return to the village. This seasonal migration is hypothesized to be one of the reasons for the cholera outbreak, as any of these migrant workers might have returned to the village, harboring the infection.

Imagine an Indian monsoon in its full fury: thunderclouds, overcast skies, and incessant pouring rain. A small isolated village surrounded by lush paddy fields, now transformed into muddy swamps. This was the setting for the outbreak reported to be due to contamination of an open well. The well, which was in use since the time of Ahilyabai Holkar, a legendary regent queen in the 18th Century, was the only source of drinking and domestic water for the village.

Rahude might be struggling for basic civic necessities, but it has a long history of settlement, evidenced by dilapidated buildings and other structures. The village is dependent on groundwater, and multiple sources have been explored. Earlier, a step well in the middle of the village served as a source of water. This was closed by health agencies as part of the dracunculiasis eradication program. In recent years, the village was dependent on two wells as sources of potable water, of which only the bigger well was presently in use. However, a high water table and the presence of a drainage channel near the well always posed a risk for contamination.

The young medical officer in charge of the area narrated her account of the outbreak. She had slept in a vehicle for a week, going home only to freshen up. The primary health center and the emergency unit setup were chockablock with patients. She had worked round the clock to manage the outbreak along with her team of doctors, nurses, and health care workers. Initially, when the health center was full, she referred patients to a government hospital about 30 km away. When she realized that the rain and flooding meant inordinate delays in travel, she took the initiative to set up an emergency health unit at the Anganwadi with the support of the district health authorities. Almost 200 patients required medical attention during that period, and she was the only clinician present in the village. Her efforts were well appreciated by the health workers of the area and the villagers.

This young lady was on temporary deputation to Rahude, and the day of our visit was also her last day in the village. Despite this, she took time out to meet us and accompanied us for the whole day. Her job was additionally challenging because of low literacy rates, local traditions, and superstitions prevalent in the community. However, unshattered by all this, we found her focused and determined to carry out her responsibilities.

The Accredited Social Health Activist (ASHA) workers reminisced about wading through waist deep water, mud, and slush to visit the houses allotted to them. Employed under the National Health Mission, an ASHA worker is selected from the village itself and accountable to it. She is trained to work as an interface between the community and the public health system. One imagines struggling through the narrow flooded lanes, with bags full of oral rehydration therapy packets, registers, and orthotoluidine test kits (Ltek Systems, Nagpur, India). Definitely not something easy to handle and yet these women, some young and some not so young, had cheerfully performed their duties with deep pride and satisfaction.

The ASHA workers have now become the key to providing medical assistance in rural populations. As female workers, ASHAs can effectively interact with female members of the families. In villages like Rahude, their entry in the household is more acceptable during the time when male members migrate to the cities and only females stay back. Accredited Social Health Activist workers are locals and aware of local tribal traditions. One ASHA worker informed us that the entire village takes part in the funeral ceremonies of the deceased. So, enquiring from people whether they have attended a funeral might not be relevant.

As public health professionals, we have for long known the inequalities between the rural and urban health-care delivery systems in India, including the shortage of qualified medical practitioners, the lack of facilities, and the limitations of infrastructure. The question which now arose was whether we are so caught up in what we lack that we fail to appreciate what we have. The experience in Rahude supported this thought. To carry out a household survey after the outbreak, we were accompanied by a team of health workers, including community health workers like ASHAs, multipurpose health workers, health assistants, a health supervisor, and auxiliary nurses and midwives—in short, the entire galaxy of rural health workers. Their organization, systematic approach, communication, and data recording skills left us wonderstruck.

When at the end of a long and hard day visiting households, conducting interviews, and recording data, we informed the team that the next day would begin at 7 am, there was not a single negative response, only cheerful assent. Surprised, we asked them if they were okay with coming early, as they were going home late as well. One bright-eyed young woman answered with a smile that they would manage. She added that, if this work could help them understand why their villagers got cholera, they would step in to prevent it happening again.

Community initiatives play a major role in the prevention and control of cholera and other water-borne diseases. We learnt that the panchayat (village local body) had been spearheading the construction of tube wells as an alternative source of water. We saw one young girl happily pumping water from a newly installed hand pump. However, the use of well water had not stopped completely, and it was being used for other domestic purposes. We learnt that women found it easier to draw water from a well than pump it from the ground. Construction of a water tank, which had been stalled, was now nearing completion, and piped water, which had remained a dream, could soon become a reality for these villagers. Moreover, we hope that the widespread illiteracy will also reduce with the new village school coming up. The school will pave the way for effective implementation of welfare programs, particularly for women and children.

As we prepared to leave for Pune, these real-life warriors against disease stepped up to us and thanked us for coming to Rahude. It was we instead who had to thank them for teaching us a valuable lesson in health care. Nothing should stop us—not lack of infrastructure, manpower, or support—from doing the very best for patients in our care.

